# The Benefits of Artemisinin Combination Therapy for Malaria Extend Beyond the Individual Patient

**DOI:** 10.1371/journal.pmed.0020105

**Published:** 2005-04-26

**Authors:** Paul Garner, Patricia M Graves

## Abstract

Garner and Graves discuss the implications of a new study in PLoS Medicine that found that artemisinin combination treatment reduces infectiousness

The traditional, low-cost mainstay drugs for malaria, chloroquine (CQ) and sulphadoxine-pyrimethamine (SP), have a very limited lifetime left in terms of their clinical usefulness. These drugs have been relatively ineffective in Asia for two decades, and rising drug resistance levels have now also rendered them ineffective in many sub-Saharan African countries [1]. Artemisinin drugs, such as artesunate and artemether, derived from the Chinese herb Artemisia annua, are rapidly being adopted as standard treatments in Africa, in the hope that effective treatment will assist in reversing the apparently increasing death rates in African children [2].

## The Impact of Artemisinin Derivatives on Gametocytes

In the most severe form of human malaria, Plasmodium falciparum, adequate treatment of a malaria attack does not necessarily prevent the infected person from transmitting the disease to others. Mosquitoes become infected by ingesting mature sexual stages, known as gametocytes, which take around ten days to develop. Mature gametocytes present at the time of treatment may be unaffected, and parasites already committed to gametogenesis at the time of treatment will continue their development. Therefore, infectious gametocytes may be present in the blood for many days after the patient has been treated and feels better. Gametocytes responsible for such “post-treatment transmission” are more likely to carry and spread drug-resistant alleles [3].[Fig pmed-0020105-g001]

**Figure pmed-0020105-g001:**
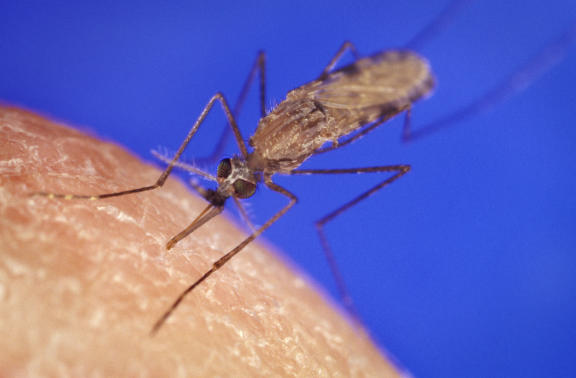
ACT reduces infectiousness to mosquitoes (Photo: Centers for Disease Control and Prevention/ James D. Gathany)

Trials showed that adding three days of the artemisinin derivative artesunate to existing antimalarial treatments led to a marked reduction in the number of gametocytes in the month following the treatment [4]. Whether this translates to an impact on transmission is less clear: in an area of low transmission in western Thailand, the introduction of artesunate in 1994 coincided with a sustained decrease in incidence of malaria [5]. However, infectiousness of patients to mosquitoes in this study was not measured directly, and the association of drug introduction and incidence reduction may have been coincidental.

A randomized controlled study by Sutherland and colleagues in this month's *PLoS Medicine* [6] shows conclusively that a six-dose course of artemether-lumefantrine (co-artemether) given to children with P. falciparum malaria in Gambia reduces gametocyte prevalence, duration of gametocyte carriage, and infectiousness to mosquitoes, compared to dual treatment with CQ and SP (CQ/SP). Presumably owing to lower gametocyte density, none of the artemether-lumefantrine-treated gametocyte carriers were infectious on day 7 after treatment, compared to 37% of the CQ/SP-treated children. The results also show that artemether-lumefantrine has a specific action against developing gametocytes in addition to its action on asexual stages. This effect of artemether-lumefantrine gives it a significant advantage over CQ/SP, although in terms of parasitological and clinical failure no difference was found between the two regimens.

## Scaling Up Artemisinin-Based Combination Treatment

Whilst there is little argument that artemisinin-based combination treatments (ACTs) such as artemether-lumefantrine are effective in treating malaria, they are a lot more expensive than the treatments currently being used. The advocates of policy change are lobbying hard for the global financing mechanisms that will likely be necessary in order to subsidize the world's supply of ACTs [7]. The fervor of the advocates is intense [8], but national policies—particularly the introduction of interventions that are in short supply and cost more to produce—are unlikely to be implemented at the speed that a clinician treating an individual patient would desire. It is more likely that countries will explore the variety of strategic options open to them, which include ACTs as first-line treatment, as well as non-artemisinin combination treatments, such as amodiaquine with SP, and will examine the potential benefits and costs [9].

With ACTs, the potential public health impact of reducing transmission is a factor to include in the evaluation of benefits versus costs, but there is little research on this impact in areas that are highly endemic for malaria. The finding of Sutherland and colleagues that ACTs also greatly reduce infectiousness will contribute to appropriate and informed decision-making for sustainable changes in treatment policy at the country level.

## Abbreviations

ACT: artemisinin-based combination treatment
CQ: chloroquine
SP: sulphadoxine-pyrimethamine
